# The Status and Prospects of Epigenetics in the Treatment of Lymphoma

**DOI:** 10.3389/fonc.2022.874645

**Published:** 2022-04-08

**Authors:** Jiaxin Liu, Jia-nan Li, Hongyu Wu, Panpan Liu

**Affiliations:** ^1^ State Key Laboratory of Oncology in South China, Collaborative Innovation Center for Cancer Medicine, Sun Yat-sen University Cancer Center, Guangzhou, China; ^2^ Department of Medical Oncology, Sun Yat-Sen University Cancer Center, Guangzhou, China

**Keywords:** epigenetics, lymphoma, DNA methylation, histone methylation, RNA methylation, histone acetylation

## Abstract

The regulation of gene transcription by epigenetic modifications is closely related to many important life processes and is a hot research topic in the post-genomic era. Since the emergence of international epigenetic research in the 1990s, scientists have identified a variety of chromatin-modifying enzymes and recognition factors, and have systematically investigated their three-dimensional structures, substrate specificity, and mechanisms of enzyme activity regulation. Studies of the human tumor genome have revealed the close association of epigenetic factors with various malignancies, and we have focused more on mutations in epigenetically related regulatory enzymes and regulatory recognition factors in lymphomas. A number of studies have shown that epigenetic alterations are indeed widespread in the development and progression of lymphoma and understanding these mechanisms can help guide clinical efforts. In contrast to chemotherapy which induces cytotoxicity, epigenetic therapy has the potential to affect multiple cellular processes simultaneously, by reprogramming cells to achieve a therapeutic effect in lymphoma. Epigenetic monotherapy has shown promising results in previous clinical trials, and several epigenetic agents have been approved for use in the treatment of lymphoma. In addition, epigenetic therapies in combination with chemotherapy and/or immunotherapy have been used in various clinical trials. In this review, we present several important epigenetic modalities of regulation associated with lymphoma, summarize the corresponding epigenetic drugs in lymphoma, and look at the future of epigenetic therapies in lymphoma.

## 1 Introduction

Lymphoma is the most common lymphoid malignancy and is among the ten most prevalent cancers worldwide. It can roughly be subclassified into Hodgkin’s lymphoma (HL) and Non-Hodgkin’s lymphoma (NHL) (1, 2). NHL accounts for about 90% of all lymphomas and the remaining 10% are referred to as HL (3). NHL is the sum of a group of independent diseases with strong heterogeneity that can be divided into B cell, T cell, and NK cell lymphomas according to the lymphocyte type. While 90% of early-stage HL patients and more than 50% of NHL patients respond to first-line conventional treatment, the remaining ones and those with relapsed disease, are still challenging to treat (4, 5).

With the deepening in the understanding of tumor pathogenesis, it has become clear that the occurrence and development of tumors is not only related to gene mutations and deletions but also to the imbalance of epigenetic regulation. In the past, it was believed that tumors were diseases driven by the accumulation of gene mutations (6). In fact, epigenetic alterations in tumors are much more frequent than the existing identified genetic alterations. The epigenetic variations are not only associated with classical signaling pathways such as those for cell growth, proliferation and apoptosis but also lead to changes of new signal transduction pathways such as those for immune escape, energy metabolism disorders, activation of cellular phenotype transition, and promotion of tumor inflammation (6–10).

The core of epigenetic changes is the covalent modification of histones and nucleic acids to determine the chromatin configuration and unique transcription spectrum in cells (11). Chromatin is formed by a DNA measuring about 2 meters in length, wound around the nucleosome composed of four histones. The total chromatin is packed into 23 pairs of chromosomes by forming a quaternary structure, which is stored in about 7 μm in the nucleus of cells (7). The most common epigenetic modifications are histone modifications and DNA methylation at the fifth carbon atom of cytosines. DNA methylation and histone deacetylation result in a dense chromatin conformation, leading to gene transcriptional silencing. On the contrary, DNA demethylation and histone acetylation lead to a loose chromatin conformation and active gene transcription. In addition to covalent modification of histones and nucleic acids, epigenetic regulation also includes dynamic spatio-temporal positioning of nucleosomes, regulation of chromatin three-dimensional conformation and nuclear topology, regulation of non-coding RNA, microRNA and enhancer RNA (12, 13). In conclusion, the action of multiple epigenetic factors influence chromatin conformation, resulting in an anomalous interaction between DNA and transcription factors, abnormal regulation of gene transcription and signaling pathways. Abnormal inactivation of signaling pathways and tumor suppressor gene pathways may lead to tumorigenesis that may provide the possibility of using existing epigenetic regulators to restore normal gene expression.

## 2 Epigenetic Targets in Lymphoma

### 2.1 DNA Methylation

#### 2.1.1 DNMT

DNA methylation is an important epigenetic mechanism in normal cells as well as tumor cells that can affect gene expression by directly controlling the activity of DNA regulatory elements, including cytosine-phosphate-guanine (CpG) islands in the promoter region (14). DNA methylation occurring at the 5-carbon of cytosine residues in CpG dinucleotides is the first characteristic of an epigenetic modification of chromatin (15). 5-methylcytosine (5mC) is produced by the transfer of methyl groups to 5-cytosine using S-adenosyl methionine (SAM) as a methyl donor under the catalysis of DNA methyltransferase (DNMTs). DNMTs are multi-domain proteins in which two functional parts can be distinguished, a large N-terminal regulatory part and a smaller C-terminal part (16, 17). The C-terminal domains of DNMTs contain 10 conserved amino acid motifs that are characteristic for specific DNA-(cytosine-C5)-MTases. They are involved in DNA recognition and binding, target base flipping and catalysis, so they are called target recognition domains (TRD) (18). The N-terminal part of DNMTs includes several regulatory domains that guide the nuclear localization of enzymes, mediate their interaction with other proteins, regulatory nucleic acids (such as non-coding RNA) and chromatin, and perform post translational modification (PTM) (15, 19). They are classified into DNMT1, DNMT3A and DNMT3B according to the N-terminal regions. DNMT1 catalyzes DNA methylation retention that maintains the genetic stability of methylation sites during replication; DNMT3A and DNMT3B catalyze the *de novo* methylation of DNA (20, 21). The expression of *DNMT1* is up regulated in mantle cell lymphoma (MCL) (22) and can be inhibited by DNMT inhibitor decitabine (23). DNMT1 and DNMT3B show *MYC*-dependent overexpression in Burkitt’s lymphoma (BL). MYC directly binds to DNMT1 and DNMT3B promoters, resulting in an increase in their transcription in the human BL model (24). All three DNMTs are overexpressed in diffuse large B-cell lymphoma (DLBCL), which is significantly correlated with advanced clinical stage and adverse reactions to chemotherapy and/or radiotherapy (25). For example, DNMT3A is overexpressed in 30% of angioimmunoblastic T-cell lymphoma (AITL) and 40% of DLBCL and is associated with reduced overall survival (OS) and event-free survival (EFS) in DLBCL patients (26). Mutations in *DNMT3A* are more common in patients with T-cell lymphoma (27–29).

#### 2.1.2 TET

Unlike the DNMT family, which catalyzes and maintains DNA methylation, the ten–eleven translocation (TET) family of α-ketoglutarate (α-KG)-dependent dioxygenases indirectly drives DNA demethylation through 5mC oxidation catalysis (30). TET1, TET2, and TET3 in the TET family can gradually oxidize 5mC to 5-hydroxymethylcytosine (5hmC), 5-formylcytosine (5fC), and 5-carboxycytosine (5caC) (13). There are two main mechanisms by which TET protein promotes DNA demethylation: a passive (replication-dependent) DNA demethylation and an active DNA demethylation. All three oxidized methylcytosines (oxi-MC) are DNA demethylation intermediates. During DNA replication, if oxi-MC exists on the template chain, unmethylated cytosine on the newly synthesized chain will not be effectively recognized or methylated by DNMT1 complex, resulting in loss of DNA methylation during cell division (31). This passive (replication-dependent) DNA demethylation is the main demethylation mechanism in most cells. Active DNA demethylation means that 5fC and 5caC can be removed from properly base-paired 5fC:G and 5caC:G base pairs by thymine DNA glycosylase, which normally excises T:G mismatches; then the base excision repair system replaces oxi-MC with unmodified cytosine (32). Among the three *TET* genes, *TET2* has repeated inactivation mutations in a wide range of bone marrow and lymphoid malignant tumors (33). *TET2* mutations include deletion, missense, nonsense, and frameshift mutations. Numerous studies have shown that most patients with AITL and peripheral T-cell lymphoma, not otherwise specified (PTCL-NOS) carry *TET2* mutations (28, 32, 34–36) and decreased OS of patients (37). Most AITL and some PTCL-NOS may come from follicular helper T (Tfh) cells, the T cells that facilitate B cell antibody responses by interacting with B cells in the germinal center (38). In AITL multistep tumor model, *TET2* and/or *DNMT3A* mutations occurred first, followed by specification into the Tfh lineage guided by expression of the *RHOA^G17V^* mutant and enhanced by hyper activation of the T-cell receptor signaling pathway (39). The expansion and/or dysfunction of Tfh can induce the production of cytokines, which play an important role in the early stage of lymphoma progression and the rich inflammatory components of AITL tumor lesions (39, 40). Similarly, TET2 mutations are common in B-cell lymphomas, especially in DLBCL (34).

#### 2.3 IDH

As mentioned earlier, TET enzyme depends on the metabolic cofactor α-KG. However, in case isocitrate dehydrogenase (IDH) is mutated, α-KG might be converted into D-2-hydroxyglutarate (D-2-HG) which blocks TET2 function. A frequent mutation in the *IDH* family is *IDH2^R172^*. The *IDH2* mutation often occurs in AITL and the D-2-HG, produced by the mutated enzyme, is a tumor metabolite (28, 41). *IDH2* mutation also affects histone lysine methylation. In AITL patients, in which the disease was caused by an *IDH2^R172^* mutation, the level of trimethylated H3 at lysine 27 (H3K27me3) increased significantly (29, 42).

#### 2.1.2 Histone Methylation

Histone methylation is catalyzed by histone methyltransferase (HMT) and occurs at different lysine and arginine of histone, which may involve monobasic, dimethyl and trimethylation at the same residue. In addition, the dimethylation of arginine can be symmetric (me2s) or asymmetric (me2a) (43). Depending on the target residue, methylation level and symmetry, methylation corresponds to different gene expression and function, which affects the level of gene transcription and leads to gene transcriptional activation or inhibition. For example, trimethylated H3 at lysine 4(H3K4me3) and dimethylated H3 at lysine 79 (H3K79me2) are beneficial to transcription, while H3K27me3 and trimethylated H3 at lysine 9(H3K9me3) inhibit transcription (43, 44).

##### 2.1.2.1 KMT2, DOT1L

Histone-lysine N-methyltransferase 2 (KMT2), which was initially named the mixed-lineage leukaemia (MLL) family, on the one hand, can directly H3K4me3 (45), on the other hand, it can change the chromatin state and DNA accessibility by recruiting demethylases to reduce H3K27me3. The KMT2 family includes KMT2A, KMT2B, KMT2C, KMT2D, KMT2F, and KMT2G. Nonsense or frameshift mutations frequently occur in DLBCL and follicular lymphoma (FL), resulting in down-regulation of KMT2D protein expression (46, 47). Zhang et al. demonstrated that FL and DLBCL-associated KMT2D mutations impair KMT2D enzyme activity, resulting in reduced global H3K4 methylation in germinal center (GC) B cells and DLBCL cells (48). Thus KMT2D is considered a tumor suppressor gene whose early deletion promotes lymphoma formation by remodeling the epigenetic landscape of cancer precursor cells. In MCL and Extra nodal NK/T-cell lymphoma, nasal type (ENKTL-NT), KMT2D mutation indicates a poor prognosis (49). KMT2D deficiency can lead to changes in a variety of genes, including *TNFAIP3* (*A20*), *SOCS3, SGK1, TRAF3, TNFRSF14 (HVEM)* and *ARID1A*, which in turn affect CD40, JAK-STAT, toll like receptor and the B-cell receptor pathway (47). Disruptor of telomeric silencing 1-like (DOT1L) is the only member of the KMT4 family. DOT1L can H3K79me2 and promote acetylation of H4, which in turn regulates the binding of bromodomain-containing protein 4 (BRD4) to chromatin (50). A potent and selective amino-nucleoside inhibitor of DOT1L histone methyltransferase activity, EPZ-5676, inhibited H3K79 methylation and MLL fusion target gene expression in cellular studies and showed selective and effective cell killing of acute leukemia lines carrying MLL translocations (51).

##### 2.1.2.2 EZH2

The function of Enhancer of Zeste Homolog 2 (EZH2) is opposite to that of KMT2. EZH2 is a HMT of 746 amino acids and is a catalytic subunit of Polycomb Repression Complex 2 (PRC2) that can inhibit gene transcription by catalytic formation of H3K27me3, can also recruit histone deacetylase (HDAC) 1/2 and DNMTs to further inhibit transcription through its cofactor embryonic ectoderm development (EED) (52, 53). EZH2 is highly expressed in GC B cells and targeted by somatic mutations in B-cell lymphomas (54). In particular, activating mutations in EZH2 are frequently found in FL and germinal center DLBCL (GC-DLBCL) (55–57). *MYC* related EZH2 overexpression has been found in BL and double hit lymphoma (58). In DLBCL and FL, EZH2 catalyzes somatic heterozygous mutations of Y641 and A677 residues in the set domain (44, 59), thereby promoting transcriptional inhibition and tumorigenesis by increasing the level of H3K27me3 (60). Many experiments have confirmed that *Ezh2^Y641^* mutation and *Myc* synergistically promote the formation of lymphoma which has been shown in transgenic mouse models (61, 62). Similar to a Y641 mutant cell line, a *EZH2^A677^* mutant cell line showed abnormal increase of H3K27me3 and decrease of monomethylated H3K27 (H3K27me1) and dimethylated H3K27 (H3K27me2) (63). For T-cell lymphoma, it is reported that in 67.5% PTCL-NOS, 50% natural killer/T-cell lymphoma (NKTCL), in 73.3% anaplastic large-cell lymphoma (ALCL), and in 60% AITL cases EZH2 was strongly expressed, these patients with peripheral T-cell lymphoma (PTCL) overexpression were often accompanied by more complications and displayed lower survival rates (64).

##### 2.1.2.3 SETDB1

SET Domain, Bifurcated 1 (SETDB1) catalyzes the trimethylation of histone H3K9 (H3K9me3) and thereby promotes transcriptional silencing (65), the N-terminal of SETDB1 interacts directly with the plant homeodomain of DNMT3A and localizes to a silent promoter in cancer cells (66). A recent study showed that simultaneous inhibition of G9a (another methyltransferase of H3K9) and DNMTs with the dual inhibitor CM-272 enhanced antitumor immunity alone or in combination with anti-PD1 (67).

##### 2.1.2.4 LSD1

Histone methylation is a dynamic equilibrium process and is a reversible histone modification. Lysine-specific demethylase 1 (LSD1/KDM1A) is a flavin adenine dinucleotide (FAD)-dependent demethylase that specifically removes monomethylated and dimethylated groups from H3K4 and H3K9 (H3K4me1, H3K4me2, H3K9me1, and H3K9me2) (68). In a mouse model, B-cell lymphoma 6 (Bcl6) was found to directly bind and recruit LSD1, and conditional deletion of *Lsd1* suppressed GC proliferation induced by constitutive expression of Bcl6 and significantly delayed Bcl6-driven lymphangiogenesis. This suggests that LSD1 plays a key role in lymphangiogenesis as an important BCL6 cofactor, as this classical lymphoma oncogene requires LSD1 to induce malignant transformation (69). *LSD1* is overexpressed in human DLBCL tissues and negatively correlates with the OS of DLBCL patients (70). *LSD1* was found to be upregulated and positively correlated with Ki67 in MCL patients, while H3K4me1 and H3K4me2 were downregulated (71).

##### 2.1.2.5 PRMT

Arginine methylation is catalyzed by protein arginine methyltransferase (PRMT), that can be sub classified into type I and type II enzymes that are responsible for the formation of asymmetric and symmetric dimethylarginines, respectively. PRMT5 is the major type II enzyme that catalyzes the symmetrical dimethylarginine of histones and induces gene silencing by generating repressive histone tags, including the arginine asymmetric dimethylation of histones H2AR3, H3R8, and H4R3 (H2AR3me2s, H3R8me2s, and H4R3me2s) (72). In the cytoplasm, PRMT5 is involved in the formation of the 20S protein arginine methyltransferase complex, which forms the “methylome”. The complex consists of the shedder Sm protein, PRMT5, pICln, and WD repeat protein (MEP50/WD45). PRMT5 methylates the Sm protein, which in turn regulates shedder activity and downstream gene expression (73). In *Eµ-myc* transgenic mice, MYC directly upregulates transcription of core small nuclear ribonucleoprotein particle assembly genes, including *Prmt5*, as a way to ensure splicing fidelity of exons with weak 5’ donor sites-an important step in lymphomagenesis (74). PRMT5 is overexpressed in MCL, GC-DLBCL, and activated B cell-like DLBCL (ABC-DLBCL) cell lines and clinical samples as well as in mouse primary lymphoma cells. PRMT5 upregulates PRC2 expression by epigenetically silencing RBL2 and indirectly causing RB1 inactivation through phosphorylation (75, 76). PRMT5 knockdown reactivates the RB1/RBL2-E2F tumor suppressor pathway and antagonizes cyclin D1-CDK4/6 signaling, which in turn leads to lymphoma cell death. Another study found that PRMT5 directly silenced the expression of axin-related protein (AXIN2) and WNT inhibitory factor 1 (WIF1). This lead to a stimulation of WNT/β-catenin signaling and indirectly activated the AKT/GSK3β pathway, leading to an inhibition of the overexpression that induced lymphoma cell death (41).

#### 2.1.3 RNA Methylation

According to the data analysis of the RNA modification database MODOMICS as of 2017, 163 different chemical RNA modifications have been identified in all organisms (77). Among them, N6-methyladenosine (m^6^A) is considered to be the most common, rich and conservative internal PTM in eukaryotic messenger RNAs (mRNAs), microRNAs (miRNAs), and long non-coding RNAs (lncRNAs). M^6^A usually occurs in adenine of the common sequence RRACH (R=A/G,H=A/C/U) (78), is enriched near the stop codon and the 3′ untranslated terminal region (UTR) and translated near the 5′ UTR in a cap-independent manner (79).

M^6^A-RNA methylation modification is a reversible biological process participated by methyltransferases (writers), demethyltransferases (erasers) and methylation readers (readers), which affects RNA transcription, processing, translation and metabolism. Writers include methyltransferase-like 3 (METTL3) (80), METTL14 (81, 82), Wilms tumor 1-associated protein (WTAP) (83), RNA-binding motif protein 15/15B (RBM15/15B) (84), KIAA1429 (85), and zinc finger CCCH-type containing 13(ZC3H13) (86); readers comprise e.g. YT521-B homologue (YTH) protein family (87), insulin-like growth factor 2 mRNA-binding proteins (IGF2BP1/2/3) (88), eukaryotic initiation factor (eIF) 3 and heterogeneous nuclear ribonucleoprotein (hnRNP) family (86); whereas erasers include fat mass and obesity-associated protein (FTO) (89) and alkB homologue 5 (ALKBH5) (86, 90, 91).

METTL3 has a SAM-binding domain which can catalyze the transfer of methyl groups in SAM to adenine bases in RNA to produce S-adenosine homocysteine (SAH), while METTL14 is mainly used to stabilize the structure of the methyltransferase complex (MTC) and to determine a specific RNA sequence (“RRACH”) as a catalytic substrate (92). Both of them were co-located in nuclear speckles and formed a stable complex at a ratio of 1:1 (81). WTAP, RBM15/15B, and KIAA1429 don’t have a catalytic function. WTAP is responsible for recruiting METTL3-METTL14 heterodimers, which form the m^6^A methyltransferase tricomplex (METTL3–METTL14–WTAP); RBM15/15B binds METTL3 and WTAP and directs these two proteins to specific RNA sites for m6A modification, which play important roles in cell growth and apoptosis, especially in blood cells, by regulating various signaling pathways such as Notch and Wnt; KIAA1429 recruits MTC and mediates methylation of adenine bases near the 3’UTR and stop codon regions in mRNA (83, 84, 86, 93). Through the interaction between ZC3H13 and WTAP, its low-complexity (LC) domain is retained in nuclear speckles, thus improving its catalytic function (94).

FTO and ALKBH5 belong to the 2-oxoglutarate-dependent nucleic acid oxygenase (NAOX) family and catalyze the demethylation of m^6^A in a Fe^2+^ and α-KG-dependent manner. The FTO in the nucleus mediates the demethylation of m^6^A, whereas the FTO in the cytoplasm mediates the N6 and the dimethyladenosine (m^6^Am) and m^6^A in the cytoplasm. In addition, FTO can also combine with transfer RNA (tRNA) to mediate the demethylation of N1-methyladenosine (m^1^A) in tRNA (95, 96). ALKBH5 colocalizes with nuclear speckles and influences mRNA processing factors’ assembly/modification and regulates mRNA export and RNA metabolism (97).

Writers play a positive catalytic role in RNA methylation modification, which can be reversed by erasers. However, in this process, different readers need to identify the modified residues and transmit information to complete the downstream biological function and establish an efficient and orderly m^6^A regulatory network. The YT521-B homology (YTH) domain family includes YTH domain family protein 1(YTHDF1), YTH domain family protein 2 (YTHDF2), YTH domain family protein 3 (YTHDF3), YTH domain containing 1(YTHDC1), and YTH domain containing 2 (YTHDC2) (98). YTHDF1/2/3 are located in the cytoplasm. The C-terminal region of YTHDF2 can identify specific m^6^A sites, and its N-terminal region binds to the SH domain of CCR4-NOT transcription complex subunit 1 (CNOT1), thereby recruiting the CCR4-NOT deadenylase complex. After this series of processes, RNA is finally transported to the processing body (P-body) to accelerate RNA degradation. YTHDF1 interacts with eukaryotic translation initiation factor 3 (eIF3), eIF4E, and eIF4G to improve the translation efficiency of m^6^A modified mRNA (86, 99); YTHDF3 promotes the translation of related mRNA through direct interaction with YTHDF2. YTHDC1 binds pre-mRNA and interacts with mRNA splicing factor, specifically recruiting serine- and arginine-rich splicing factor 3 (SRSF3) or antagonizing serine- and arginine-rich splicing factor 10 (SRSF10). Thereby promoting exon inclusion, splicing, as well as mRNA export from the nucleus to the cytoplasm (100). YTHDC2 selectively binds m^6^A at its consensus motif, enhances the translation efficiency of its targets and also decreases their mRNA abundance (101). The YTH family is the most important type of m^6^A readers, however, additional proteins are involved in this processing cascade. For example, IGF2BP1/2/3 rely on their K homology (KH) domains to recognize consensus GG (m^6^A) C sequences, promote the stability and storage of their target mRNAs in an m^6^A-depedent manner under normal and stress conditions and thus affect gene expression output (88). HNRNPA2B1 can bind to m^6^A-bearing sites in the transcriptome and positively regulates primary miRNA transcript (pri-miRNA) processing in a similar manner as METTL3 (102).

In lymphoma, m^6^A in DLBCL was studied the most. In DLBCL tissues and cell lines, the expression of METTL3 is up-regulated, which leads to the increase of the m^6^A level of pigment epithelium-derived factor (PEDF) expression and transcription, and finally resulting in the activation of the Wnt pathway which accelerates cell proliferation. Down-regulation of METTL3 expression can inhibit the proliferation of DLBCL cells (103). Both knockdown and overexpression of METTL3 protein will lead to the upregulation of WTAP protein. The level of METTL3 is closely related to the homeostasis of WTAP, and in the absence of METTL3, the upregulation of WTAP is not enough to promote cell proliferation (104). Therefore, we speculate that WTAP plays a carcinogenic role in DLBCL and may be closely related to m^6^A-RNA methylation co-participated by METTL3. WTAP forms a complex with heat shock protein 90 (HSP90) and BCL6 to maintain its stability, thus promoting the proliferation of DLBCL cells and improving the ability to resist apoptosis. After the use of the antineoplastic drug etoposide in a DLBCL cell line, the expression of WTAP decreased and the apoptosis rate of tumor cells increased significantly (105). Another study showed that WTAP enhances the hexokinase 2 (HK2) m^6^A level by enhancing the expression of the *HK2* gene, a process regulated by PIWI-interacting RNAs (piRNAs)-30473 (106, 107). HK2 is the rate limiting enzyme of the glycolysis pathway which can enhance aerobic glycolysis and promote tumor cell proliferation. Previous studies have confirmed that HK2 is the key metabolic driver of the DLBCL phenotype (108). In addition, WTAP was obviously upregulated in human NKTCL cell lines (YTS and SNK-6 cells), compared with normal NK cells. More importantly, intervention of WTAP evidently prohibited NKTCL cell chemotherapy resistance to cisplatin (109).

Wu et al. found that MYC activates the expression of ALKBH5 and YTHDF3, reducing m^6^A levels in the mRNA of the selected MYC-repressed genes (MRG) *SPI1* and *PHF12*. By inhibiting ALKBH5, or overexpression of SPI1 or PHF12, effectively suppresses the growth of MYC*-*deregulated B-cell lymphomas, both *in vitro* and *in vivo (* 110). In addition, whole-exome sequencing (WES) showed deletions and mutations of YTHDF2 in PTCL (29). It was shown that Ki-67-related IGF2BP3 is the most strongly upregulated mRNA in MCL cases, and its high expression is closely related to the proliferation ability of tumor cell (111). In Zhang’s study, 10 m^6^A modulators were classified according to the risk ratio to predict the survival rate of patients with MCL (38).

#### 2.1.4 Histone Acetylation

Chromatin histone acetylation and deacetylation are also key steps in epigenetic regulation. These two reversible processes are jointly regulated by histone acetyltransferase (HAT) and HDAC and are in dynamic equilibrium under normal physiological conditions (43).

##### 2.1.4.1 HAT

HATs use acetyl coenzyme A as a cofactor and catalyze the transfer of acetyl groups to the ϵ-amino group of the lysine side chain. This leads to the neutralization of the positive lysine charge and thus potentially weakens the electrostatic interaction between the histone and the negatively charged DNA, which finally results in a more “open” chromatin conformation (112). HATs are classified into type A and B. Type A HATs are located in the nucleus and are capable of modifying histones adulterated in chromatin. They are a very diverse family of enzymes that can be divided into three separate families: GNAT, MYST, and CREBBP/EP300 family (113). All of them not only modify multiple sites in the N-terminal tail of histones, but also acetylate the globular histone core (112). By establishing a mouse model, *Crebbp* and *Ep300* were found to be frequently mutated in B-cell lymphomas, mainly in DLBCL and FL (114). *CREBBP* mutations were found in 15-30% of DLBCL and 40% of FL, while *EP300* mutations were found in approximately 5% to 10% of DLBC and FL (115, 116). Mutations in HATs occur in a single allele and this mutation leads to inactivation of the HAT coding domain, which in turn affects on the one hand the acetylation of histones and non-histones, and on the other hand activates *BCL6* and the tumor suppressor *P53* involved in the development of B-cell lymphoma (117–119). *CREBBP* mutations were also found in 26% of patients with Sézary syndrome (SS) and primary cutaneous diffuse large B-cell lymphoma-leg type (PCLBCL-LT) (120, 121). Type B HATs are highly conserved and mainly acetylate free histones in the cytoplasm, but not acetylated and nucleosomal histones. Type B HATs rapidly acetylates newly synthesized histones H3 and H4, and this acetylation pattern is important for histone deposition. Moreover these modifications are removed during chromatin maturation (122).

##### 2.1.4.2 HDAC

HDACs counteract the action of HATs and reverse lysine acetylation, restoring the positive charge of lysine which may facilitate the stabilization of local chromatin structure. In humans, there are 18 HDACs that can be divided into four classes: class I Rpd3-like proteins (HDAC1/2/3 and HDAC8), class II Hda1-like proteins (HDAC4-7, HDAC9, and HDAC10), class III Sir2-like proteins (SIRT1-7), and class IV protein (HDAC11) (123). Classes I, II, and IV HDACs are zinc dependent, while class III ones are sirtuins using NAD^+^ as a reactant to deacetylate the acetyl lysine residue of the protein substrate to form nicotinamide, the deacetylation product and the metabolite 2’-O-acetyl-ADP-ribose (123, 124). The deacetylation of HDACs not only alters transcription but also other PTM such as methylation, ubiquitination and sumoylation. 55.8% PTCL-NOS, 57.1% NKTCL, 86.7% ALCL, and 50% AITL strongly expressed HDAC1; 58.1% PTCL-NOS, 57.1% NKTCL, 53.3% ALCL, and 60% AITL strongly expressed HDAC2 (64). As mentioned previously, *CREBBP* mutations disable acetylation and simultaneously enhance deacetylation of the HDAC3 complex, which may be the mechanism of GC lymphoma development (117). HDAC6 is either weakly expressed or undetectable in 96% of DLBCL cases (125) and HDAC6 may be an important prognostic marker associated with a good outcome in DLBCL or a more aggressive course in PTCL, respectively (126). HDAC7 has anti-cancer effects and expression is downregulated in BL (127). Increased *HDAC9* copy number was found in 50% of DLBCL cases and further genetic mouse models suggest that HDAC9 may contribute to lymphoma development by altering pathways related to growth and survival as well as regulating BCL6 activity and P53 tumor suppressor function (128).

##### 2.1.4.3 BET

Bromodomain and extra terminal motif (BET) family is a reader used to detect acetylated lysine residues on histones and non-histone proteins. The BET family consists of BRD2, BRD3 and BRD4, which are widely expressed in tissues, and bromodomain testis-specific protein, which is mainly found in the testis (129). The BET protein consists of two amino-terminal bromodomains that bind to acetylated lysine residues of histones and other proteins, and an extra-terminal domain, which mediates further protein-protein interactions (130). BET acts as a chromatin “reader”, transforming the chromatin state into a chromosome state by recruiting transcriptional regulatory complexes to their binding sites. In DLBCL, BL, and MCL, this action is always mediated by *MYC (* 131). For example, BRD4 interacts with and activates positive transcription elongation factor-b (P-TEFb), which stimulates RNA Pol II into active elongation and activates transcription initiation and elongation (13, 131, 132).

### 2.2 Epigenetic Therapy

**Figure 1:**
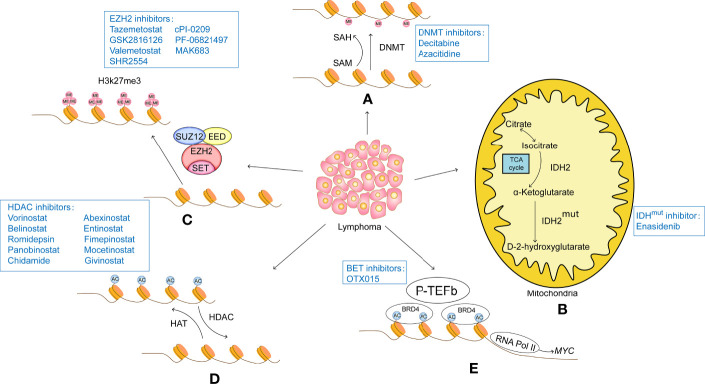
**(A)** DNA methylation modifications usually turn off gene expression and therefore result in a lack of expression of tumor suppressors. Therefore, intervention with DNA methylesterase inhibitors can reduce the methylation level of the promoter region of the target gene, opening up the expression of these tumour suppressors and thus acting as a tumour suppressor. DNA methylesterase inhibitors that have been successfully marketed include azacitidine and decitabine, both of which are nucleoside analogues that cause genome-wide reductions in methylation levels and activate gene transcription. **(B)** IDH2 is the rate-limiting enzyme of the tricarboxylic acid cycle involved in cellular energy metabolism. Under normal conditions, IDH2 catalyzes the oxidation of isocitrate to produce a-KG. Mutant IDH2 loses its normal function and converts a-KG to D-2-HG. The accumulation of D-2-HG leads to histone hypermethylation. IDH2 inhibitors such as Enasidenib target mutant IDH2 to reduce D-2-HG, thereby inducing histone demethylation and slowing tumour progression. **(C)** Histone methylation modifications are highly site-specific and modifier-specific, and have very different effects on gene expression. EZH2 is the core component of PRC2, which acts as a histone methyltransferase to catalyse H3K27me3, causing tight binding of histones to DNA and inhibiting transcription of target genes, EZH2 inhibitors such as tazemetostat, GSK2816126, valemetostat, SHR2554, cPI-0209, PF-06821497 and MAK683 specifically act on EZH2, inhibiting its function and restoring transcription of oncogenes. **(D)** Histone acetylation is regulated by HAT and HDAC. HAT catalyzes the transfer of acetyl groups to the lysine side chain of histones, which neutralizes the positively charged lysine and weakens the affinity of histones for negatively charged DNA, loosening the structure of histones and facilitating the recruitment of transcription factors and the transcription of related genes. The HDAC-catalyzed deacetylation restores the positive electrical properties of histones, resulting in a stronger electrical interaction between histones and DNA, which acts as a repressor of gene expression. A number of HDAC inhibitors have been approved for marketing, among which, vorinostat and belistat of the hydroxamic acid class were approved by the US FDA for the treatment of CTCL and PTCL in 2006 and 2014 respectively; romidepsin of the cyclic tetrapeptide class was approved by the US FDA for the treatment of CTCL and PTCL in 2009 and 2011 respectively; and chidamycin of the benzylamine class was approved by the Chinese In addition, other HDAC inhibitors, such as panobinostat, abexinostat, entinostat, fimepinostat, mocetinostat and givinostat, are also in active clinical trials. **(E)** The BET family of proteins is an important class of proto-oncoproteins that contains the bromodomain, a histone acetylation recognition factor, and a member of the BET family, BRD4, which interacts with and activates positive transcription elongation factor-b (P-TEFb) to stimulate RNA Pol II into active elongation, activating transcription initiation and elongation. The BET inhibitor competes with the acetylation residues to bind to the bromine domain of BRD4, destabilizing the DNA repair machinery and inducing the accumulation of DNA changes until cell death.

In the context of this complex epigenetic regulation of gene expression in tumors, the use of epigenetic therapies to reverse this aberrant gene expression can be effective in treating tumors. The development and testing of anti-tumor drugs targeting epigenetic factors is flourishing internationally, and a number of epigenetic drugs have been approved as drugs by the US Food and Drug Administration (FDA) in lymphoma (**Figure 1**).

#### 2.2.1 DNMT Inhibitors

##### 2.2.1.1 Decitabine

DNA demethylating agents, such as decitabine and azacitidine, have been approved by the U.S. Food and Drug Administration for the clinical treatment of myelodysplastic syndrome (MDS) and acute myeloid leukemia (AML). Decitabine is a deoxyribonucleoside that can be incorporated into DNA and occupy DNMTs to induce DNA hypomethylation. It displays cytotoxicity at high concentrations, whereas low doses can minimize toxicity and may improve the targeting effect of DNA hypomethylation through a re-expression of tumor suppressor genes during tumor therapy (133). A phase 4 clinical trial investigated the efficacy of a combination of decitabine together with a modified regimen of cisplatin, cytarabine, and dexamethasone (DHAP) in relapsed/refractory DLBCL (r/r DLBCL) (134). The results showed that overall response rate (ORR) reached 50% and complete response rate (CRR) reached 35%. Five patients (25%) showed a stable disease (SD) with a disease control rate (DCR) of 75% and the median progression-free survival (PFS) was 7 months. A randomized phase 2 study of anti-PD-1 camrelizumab plus decitabine in relapsed/refractory HL (r/r HL) achieved 79% ORR and prolonged the median PFS to 35.0 months (135). Many clinical trials are currently exploring the therapeutic efficacy of decitabine in combination with the HDAC inhibitor cidabendiamide in HL (NCT04514081, NCT04233294). The effectiveness of camrelizumab in combination with decitabine in HL (NCT04510610) and NHL (NCT04337606) is also being evaluated. There has also been an explosion in the combination of novel CAR-T therapies with the traditional epigenetic drug decitabine. For example, decitabine-primed tandem CD19/CD20 CAR-T cells treatment in relapsed/refractory B-cell NHL (r/r B-cell NHL) (NCT04697940), sequential low-dose decitabine with PD-1/CD28, CD19 CAR-T in relapsed/refractory B-cell lymphoma (r/r B-cell lymphoma) (NCT04850560). Completed or all ongoing trials are listed in **Table 1**. From these trials we can look forward to DNA demethylating agents that show potential in the field of lymphoma therapy, such as in combination with immune checkpoint agents might being regimens that can improve ORR.

**Table 1:** Clinical trials of DNA methyltransferase inhibitors.

Regimen	Disease	n	Phases	Status	Clinical Result	Survival Benefit	NCT ID
Decitabine, Cisplatin, Cytarabin, Dexamethasone (134)	r/r DLBCL	21	Phase 4	completed	50% ORR, 25% SD	The median PFS was 7 months, one-year OS rate was 59.0%, two- year OS rate was 51.6%	NCT03579082
Decitabine, Camrelizumab (135)	r/r HL	61	Phase 2	completed	79% CRR	63% maintained a response at 24 months, the median PFS was 35.0 months	NCT02961101
Decitabine, Chidamide, Camrelizumab	HL		Phase 2	Recruiting			NCT04514081
Decitabine, Camrelizumab	HL		Phase 2/3	Recruiting			NCT04510610
Decitabine, Chidamide, Camrelizumab	HL		Phase 2	Recruiting			NCT04233294
Decitabine, SHR-1210	HL		Phase 2	Recruiting			NCT03250962
Decitabine, Chidamide, Camrelizumab	NHL		Phase 1/2	Recruiting			NCT04337606
Decitabine, Sintilimab	ENTKL		Phase 2	Recruiting			NCT04279379
Decitabine, Durvalumab, Pralatrexate, Romidepsin	T-Cell Lymphoma,		Phase 1/2	Recruiting			NCT03161223
Decitabine, Pembrolizumab, Pralatrexate	PTCL, CTCL		Phase 1	Not yet recruiting			NCT03240211
Decitabine, Cyclophosphamide, Rituximab, Doxorubicin, Vincristine, Prednisone, Ibrutinib, Lenalidomide, Chidamide	DLBCL		Phase 2	Recruiting			NCT04025593
Decitabine, Rituximab, Cyclophosphamide, Doxorubicin, Vincristine, Prednisone	DLBCL		Phase 1/2	Active, not recruiting			NCT02951728
Decitabine, CD19 PD-1/CD28 CAR-T	r/r DLBCL		Phase 1	Recruiting			NCT04850560
Decitabine, CD19/20 CAR-T	r/r B-cell NHL		Phase 1/2	Recruiting			NCT04697940
Decitabine, Chidamide, CD19/20 CAR-T	r/r B-cell NHL		Phase 1/2	Recruiting			NCT04553393
Azacitidine, Vorinostat	r/r DLBCL	18	Phase 1/2	completed	6.7% ORR		NCT01120834
Azacitidine, CHOP (2021 ASH Oral No.138)	PTCL	17		completed	88.2% CRR	Two-year OS rate was 75.6%, two-year PFS rate was 69.2%	
Azacitidine, Duvelisib	Lymphoma		Phase 1	Recruiting			NCT05065866
Azacitidine, Tucidinostat, CHOP	T-cell Lymphoma		Phase 3	Not yet recruiting			NCT05075460
Azacitidine, Cyclophosphamide, Doxorubicin, Vincristine, Prednisone, Etoposide, Duvelisib	T-cell Lymphoma		Phase 2	Recruiting			NCT04803201
Azacitidine, Durvalumab, Pralatrexate, Romidepsin	T-cell Lymphoma		Phase 1/2	Recruiting			NCT03161223
Azacitidine, Romidepsin, Gemcitabine	T-cell Lymphoma		Phase 3	Active, not recruiting			NCT03703375
Azacitidine, CHOP	T-cell Lymphoma		Phase 2	Active, not recruiting			NCT03542266
Azacitidine, Duvelisib, Romidepsin, Doxorubicin	T-cell Lymphoma		Phase 1	Not yet recruiting			NCT04639843
Azacitidine, Romidepsin, Bendamustine, Gemcitabine	r/r T-cell Lymphoma		Phase 3	Active, not recruiting			NCT03593018
Azacitidine, Tislelizumab, Lenalidomide, Etoposide, Pegaspargase	NKTCL-NT		Not Applicable	Recruiting			NCT05058755
Azacitidine, Dexamethasone, Pegaspargase, Tislelizumab	NKTCL		Phase 2	Not yet recruiting			NCT04899414
Azacitidine, Vorinostat	ENTKL-NT		Phase 1	Active, not recruiting			NCT00336063
Azacitidine, Romidepsin, Belinostat, Pralatrexate, Gemcitabine	PTCL		Phase 2	Recruiting			NCT04747236
Azacitidine, Chidamide	PTCL		Phase 2	Recruiting			NCT04480125
Azacitidine, Sintilimab, Chidamide	PTCL		Phase 2	Not yet recruiting			NCT04052659
Azacitidine, Romidepsin, Lenalidomide, Dexamethasone	PTCL, CTCL		Phase 1	Recruiting			NCT04447027
Azacitidine, Bendamustine, Piamprizumab	B-cell NHL		Phase 1/2	Recruiting			NCT04897477
Azacitidine, Cyclophosphamide, Doxorubicin Hydrochloride, Prednisone, Rituximab, Vincristine Sulfate	DLBCL		Phase 2/3	Recruiting			NCT04799275
Azacitidine, Lenalidomide, Obinutuzumab	r/r B-cell Lymphoma		Phase 1	Recruiting			NCT04578600
Azacitidine, Venetoclax, Obinutuzumab	FL		Phase 1/2	Recruiting			NCT04722601
Azacitidine, R-GDP	DLBCL, r/r NHL		Phase 2	Not yet recruiting			NCT03719989
Azacitidine, R-ICE	DLBCL		Phase 1	Active, not recruiting			NCT03450343

##### 2.2.1.2 Azacitidine

Azacitidine is an analog of cytidine, which can replace nucleosides in DNA and RNA and covalently bind to DNMT to inhibit DNA methylation. The efficacy of azacitidine in the treatment of myelodysplasia is well known. A phase 1/2 study of azacitidine in combination with vorinostat in patients with r/r DLBCL resulted in a 6.7% ORR (NCT01120834). The regimen of azacitidine in combination with cyclophosphamide, doxorubicin, vincristine, and prednisone (CHOP) for PTCL-Tfh was presented at the 2021 Annual Meeting of the American Society of Hematology (ASH). Patients in the study received 300 mg azacitidine orally for 7 days before circle 1 and 14 days before circle 2-6 for a total of 6 cycles. This combination therapy achieved 88.2% CRR. In 17 Tfh patients, two-year OS and PFS reached 75.6% and 69.2%, respectively. A common grade ≥3 adverse event (AE) was neutropenia (2021 ASH Oral No.138). We also look forward to the performance of azacitidine in more lymphoma treatment cases. In the treatment of PTCL, different azacitidine combination therapy programs are in progress, such as azacitidine, romidepsin, belinostat, pralatrexate and emcitabine combined treatment protocols (NCT04747236), azacitidine and chidamide combined treatment (NCT04480125). Relevant clinical trials have been listed in **Table 1**.

#### 2.2.2 IDH2 Inhibitor

Enasidenib (AG-221) is an inhibitor of IDH2 mutations and has been approved for the treatment of AML. There is a phase 1/2 clinical trial of orally administered enasidenib (AG-221) in adults with AITL, displaying an IDH2 mutation (NCT02273739). However, the experimental results are not satisfactory. All AITL patients showed disease progression or died and had ≥ 1 treatment-emergent adverse event (TEAE).

#### 2.2.3 EZH1/2 Inhibitors

##### 2.2.3.1 Tazemetostat

On January 23, 2020, tazemetostat, the world’s first EZH2 inhibitor, was approved by the FDA to treat patients aged 16 and over with metastatic or locally advanced unresectable epithelioid sarcoma. In a phase 1 trial, monotherapy with tazemetostat showed anticancer activity and a favorable safety profile in patients with relapsed/refractory NHL (r/r NHL) (136). In this trial, 38% of the patients with B-cell NHL had an objective response and the median duration of response (DOR) was 12.4 months. In another open-label, single-arm, multicenter, phase 2 trial, tazemetostat showed a good effect in treating patients with relapsed/refractory FL (r/r FL) (137). Patients were categorised by their EZH2 status: mutant (EZH2^mut^) or wild type (EZH2^WT^). The ORR was 69% (31 of 45 patients) in the EZH2^mut^ cohort and 35% (19 of 54 patients) in the EZH2^WT^ cohort and the median PFS was 13.8 months and 11.1 months, respectively. Secondary results of another phase 1 study showed that the ORR was only 15.4% of 13 subjects with B-cell lymphoma treated with tazemetostat which may be due to excessive (69.2%) missing data (NCT03028103). Tazemetostat monotherapy has shown satisfying results so far, but we hope to see effects of tazemetostat in combination with other therapies in the treatment of lymphoma as well. Many clinical trials, as listed in **Table 2**, are exploring the effect of tazemetostat combined with monoclonal antibodies, such as ublituximab, umbralisib (NCT05152459) or rituximab (NCT04224493) in r/r FL patients.

**Table 2:** Clinical trials of EZH1/2 inhibitors.

Regimen	Disease	n	Phases	Status	Clinical results	Survival benefit	NCT ID
Tazemetostat (136)	B-cell NHL	21	Phase 1/2	Completed	38% ORR	The median DOR was 12.4 months	NCT01897571
Tazemetostat (137)	r/r FL	99	Phase 2	Completed	EZH2^mut^: 69% ORR; EZH2^WT^: 35% ORR	EZH2^mut^: the median PFS was 13.8 months; EZH2^WT^: the median PFS was 11.1 months	NCT01897571
Tazemetostat, Fluconazole, Omeprazole, Repaglinide	B-cell Lymphoma		Phase 1				NCT03028103
Tazemetostat, Ublituximab, Umbralisib	r/r FL		Phase 1/2	Not yet recruiting			NCT05152459
Tazemetostat	FL		Phase 2	Recruiting			NCT04762160
Tazemetostat, Placebo, Lenalidomide, Rituximab	r/r FL		Phase 3	Recruiting			NCT04224493
Tazemetostat, CC-99282, Rituximab, Obinutuzumab, Tafasitamab,	NHL		Phase 1	Recruiting			NCT03930953
Tazemetostat	r/r B-cell NHL		Phase 2	Active, not recruiting			NCT03456726
Tazemetostat	NHL		Phase 2	Active, not recruiting			NCT03213665
Tazemetostat, Ensartinib, Erdafitinib, Larotrectinib, Olaparib, Palbociclib, Samotolisib, Selpercatinib, Selumetinib, Sulfate, Tipifarnib, Ulixertinib, Vemurafenib	NHL		Phase 2	Recruiting			NCT03155620
Tazemetostat, Rituximab, Cyclophosphamide, Vincristine, Doxorubicin, Prednisolone	DLBCL, FL		Phase 1/2	Recruiting			NCT02889523
Tazemetostat	DLBCL, FL		Phase 2	Active, not recruiting			NCT02875548
GSK2816126 (138)	DLBCL, FL, MZL	20	Phase 1	completed	38% ORR		NCT02082977
Valemetostat	B-cell Lymphoma		Phase 2	Recruiting			NCT04842877
Valemetostat	T-cell Lymphoma		Phase 2	Recruiting			NCT04703192
Valemetostat	T-cell Lymphoma		Phase 2	Active, not recruiting			NCT04102150
SHR2554, SHR1701	Lymphoma		Phase 1/2	Recruiting			NCT04407741
CPI-0209, Irinotecan	DLBCL, T-cell Lymphoma		Phase 1/2	Recruiting			NCT04104776
PF-06821497	FL		Phase 1	Recruiting			NCT03460977
CPI-1205	B-Cell Lymphoma		Phase 1	Completed			NCT02395601
MAK683	DLBCL		Phase 1/2	Recruiting			NCT02900651

##### 2.2.3.2 GSK2816126

GSK2816126 is a potent, highly selective, SAM-competitive, small-molecule inhibitor of EZH2 methyltransferase that decreases global H3K27me3 levels and reactivates silenced PRC2 target genes. In the proliferation assay using a group of B-cell lymphoma lines, those DLBCL origins with *Ezh2* activation mutations were the most sensitive to GSK2816126 (59). The ORR in a dose-escalation phase 1 study with tazemetostat was 38% in patients with B-cell lymphomas. One of these patients with germinal centre B-cell like DLBCL (GCB-DLBCL) treated with 1,800 mg dose had a partial response lasting 91 days, and 6 patients achieved SD (5 DLBCL and 1 FL) (138).

##### 2.2.3.3 Valemetostat

Kagiyama et al. assessed the effect of a novel EZH1/2 dual inhibitor, named OR‐S1, a close analog of valemetostat, also known as DS‐3201 or (R)‐OR‐S2, on MCL tumor growth (139). In the mouse model, oral OR-S1 inhibited ibrutinib‐resistant MCL tumor growth in patient‐derived xenograft (PDX). Cyclin Dependent Kinase Inhibitor 1C (CDKN1C, also known as p57, KIP2) is a direct target of EZH1/2. OR-S1, through upregulation of CDKN1C, sharply inhibited cell proliferation which was accompanied by cell cycle arrest and B‐cell differentiation. Valemetostat is being evaluated for its effectiveness in human lymphoma. Two phase 2 trials are evaluating valemetostat monotherapy in T-cell lymphoma (NCT04703192) and B-cell lymphoma (NCT04842877) (**Table 2**).

##### 2.2.3.4 SHR2554, cPI-0209, PF-06821497, and MAK683

The therapeutic effect of other EZH2 inhibitors on lymphoma is still under further exploration. As shown in **Table 2**, a phase 1/2 study of SHR2554 in combination with SHR1701 in patients with B-cell lymphomas (NCT04407741), a study of cPI-0209 in patients with lymphoma (NCT04104776), PF-06821497 treatment of FL (NCT03460977), a study evaluating CPI-1205 in patients suffering from B-cell lymphoma (NCT02395601). A trial to evaluate the safety and efficacy of the EZH2 cofactor EED inhibitor MAK683 in DLBCL is also being recruited (NCT02900651).

#### 2.2.4 HDAC Inhibitors

HDAC inhibitors can be classified into four categories based on their chemical structure: hydroxamate, short-chain fatty acid (carboxylate), benzamide, and cyclic peptide. Among them, hydroxamate acid-based vorinostat (SAHA) and belistat were approved by the FDA for the treatment of cutaneous T-cell lymphoma (CTCL) and PTCL in 2006 and 2014, respectively; cyclic tetra peptide-based romidepsin was approved by the FDA for the treatment of CTCL and PTCL in 2009 and 2011, respectively.

##### 2.2.4.1 Vorinostat

Vorinostat is a pan-HDAC inhibitor that has been shown to cause growth arrest and cystein-dependent apoptosis as well as cystein-independent autophagic cell death with an ORR of 29.7% in a phase 2B study of 74 patients with refractory CTCL (140). This led to FDA approval of vorinostat for CTCL in 2006. Compared to total skin electron beam therapy (TSEBT) monotherapy, the combination therapy of vorinostat together with TSEBT showed a dramatically better effect (100% ORR) in mycosis fungoide (NCT01187446). Vorinostat monotherapy has also been used to treat relapsed/refractory indolent B-cell NHL and MCL. In this phase 2 trial, 56 patients were recruited and 50 were available for ORR assessment with an ORR of 44% and a median PFS of 18 months. In 39 FL patients, the ORR reached 49% and the median PFS was 20 months. The primary toxicities were manageable grade 3/4 thrombocytopenia and neutropenia (141). More clinical trials have focused on the effects demonstrated by vorinostat in combination with other drugs in the treatment of lymphoma. Vorinostat combined with aurora kinase A inhibitor alisertib (MLN8237) in relapsed/refractory lymphoid malignancy showed that of the 34 patients included, two patients with DLBCL achieved durable complete response (CR) and two patients with HL achieved partial response (PR) (142). In a trail of vorinostat combined with gemcitabine, busulfan, and melphalan with autologous stem cell transplantation in patients with refractory lymphomas, the ORR among 28 patients with DLBCL and measurable disease was 96% (143). Treatment of patients suffering from indolent NHL with a combination of vorinostat together with rituximab, demonstrated a 46% ORR and a PFS of 29.2 months (144). In a phase 2 study of vorinostat for the treatment of FL, marginal zone lymphoma (MZL) or MCL, the ORR was 29%. The median PFS was 15.6 months for patients with FL, 5.9 months for MCL, and 18.8 months for MZL (145). Vorinostat, cladribine, and rituximab were used for treating patients with MCL, relapsed chronic lymphocytic leukemia (CLL), or relapsed B-cell NHL resulted in a 79% ORR and the median PFS for relapsed NHL and previously untreated MCL was 19.5 months and 84 months, respectively (146). Vorinostat monotherapy of DLBCL was not effective with only one patient that displayed a prolonged SD of 18 evaluable patients (147). Similarly, the effect of azacitidine combined with vorinostat for the treatment of DLBCL, was not satisfactory, with only 1 out of 15 patients achieving objective response (NCT01120834). In a trial of vorinostat in combination with cyclophosphamide, etoposide, prednisone, and rituximab for elderly patients with relapsed DLBCL, the results were reasonably good with the ORR reaching 32% (NCT00667615).

The trials currently being recruited are all combination therapies of vorinostat. A phase 2 trial is exploring the role of vorinostat, gemcitabine, clofarabine, busulfan combination therapy in the treatment of NHL (NCT04220008). A phase 1 trial is investigating the effectiveness of a combination therapy of vorinostat, other chemotherapy and biological drugs in lymphoma (NCT03259503, NCT00972478, and NCT01193842). A combination azacitidine and vorinostat therapy for ENKTL is also being recruited (NCT00336063). Given the role of vorinostat monotherapy in CTCL, we believe that additional clinical trials will reveal the effectiveness of vorinostat combination therapy in other types of lymphoma. All the clinical trials mentioned above are shown in **Table 3**

**Table 3:** Clinical trials of HDAC inhibitors.

Regimen	Disease	n	Phases	Status	Clinical results	Survival benefit	NCT ID
Vorinostat (140)	CTCL	74	Phase 2	Completed	29.7% ORR		NCT00091559
Vorinostat, Total skin electron beam therapy (TSEBT)	CTCL	28	Phase 1/2	Terminated	100% ORR	Duration of clinical benefit was 28 months	NCT01187446
Vorinostat (141)	Lymphoma	50	Phase 2	Completed	44% ORR	The median PFS was 18 months	NCT00875056
Vorinostat, Alisertib (142)	Lymphoma	34	Phase 1	Completed	12% ORR		NCT01567709
Vorinostat, Gemcitabine, Busulfan, Melphalan (143)	Lymphoma	78	Phase 1	Completed		DLBCL: the EFS rate was 61.5%, the OS rate was 73%; HL: the EFS rate was 40%, the OS rate was 80%	NCT01421173
Vorinostat, Rituximab (144)	NHL	30	Phase 2	Completed	46% ORR	The median PFS was 29.2 months	NCT00720876
Vorinostat (145)	FL, MZL, MCL	35	Phase 2	Completed	29% ORR	FL: the median PFS was 15.6 months; MCL: the median PFS was 5.9 months; MZL: the median PFS was 18.8 months	NCT00253630
Vorinostat, Cladribine, Rituximab (146)	relapsed B-cell NHL	57	Phase 2	Completed	79% ORR	The median PFS was 19.5 months	NCT00764517
Vorinostat, Azacitidine	DLBCL	15	Phase 1/2	Completed	6.7% ORR		NCT01120834
Vorinostat, Cyclophosphamide, Etoposide, Prednisone, Rituximab	r/r DLBCL	30	Phase 1/2	Completed	32% ORR		NCT00667615
Vorinostat, Gemcitabine, Clofarabine, Busulfan	NHL		Phase 2	Not yet recruiting			NCT04220008
Vorinostat, Busulfan, Gemcitabine, Melphalan, Olaparib, Rituximab	r/r Lymphoma		Phase 1	Recruiting			NCT03259503
Vorinostat, Pembrolizumab	r/r NHL		Phase 1	Recruiting			NCT03150329
Vorinostat, Cyclophosphamide, Doxorubicin Hydrochloride, Etoposide, Prednisone, Rituximab, Vincristine Sulfate	B-cell Lymphoma		Phase 1/2	Active, not recruiting			NCT01193842
Vorinostat, Cyclophosphamide, Doxorubicin Hydrochloride, Prednisone, Rituximab, Vincristine Sulfate	DLBCL		Phase 1/2	Active, not recruiting			NCT00972478
Vorinostat, Azacitidine	ENKTL-NT		Phase 1	Active, not recruiting			NCT00336063
Belinostat (148)	r/r PTCL	120	Phase 2	Completed	25.8% ORR	The median PFS was 1.6 months, OS was 7.9 months,	NCT00865969
Belinostat (149)	r/r CTCL, r/r PTCL	53	Phase 2	Terminated	PTCL: 25% (6/24) ORR; CTCL: 14% (4/29) ORR		NCT00274651
Belinostat, CHOP (150)	PTCL	23	Phase 1	Completed	86% ORR		NCT01839097
Belinostat	DLBCL, BL	22	Phase 2	Completed			NCT00303953
Belinostat, Azacitidine, Romidepsin, Pralatrexate, Gemcitabine	PTCL		Phase 2				NCT04747236
Romidepsin (151)	CTCL	96	Phase 2	Completed	34% ORR	The median time to response was 2 months; the median DOR was 15 months	NCT00106431
Romidepsin (151)	CTCL	30	Phase 2	Completed	Tumor stage: 45% ORR; folliculotropic mycosis fungoides: 60% ORR	Tumor stage: the median TTR was 1.9 months; folliculotropic mycosis fungoides: the median TTR was 2.1 months	NCT00106431
Romidepsin (152)	PTCL	45	Phase 2	Completed	38% ORR	The median DOR was 8.9 months	NCT00007345
Romidepsin (153, 154)	PTCL	130	Phase 2	Completed	25% ORR	The median PFS was 4 months; OS was 11.3 months	NCT00426764
Romidepsin (155)	PTCL	40	Phase 2	Completed	43% ORR	The median PFS was 5.6 months; the median DOR was 11.1 months	NCT01456039
Romidepsin (156)	PTCL	18	Phase 3	Completed	43% ORR	The median PFS was 242 days	NCT01482962
Romidepsin, Azacitidine (157)	PTCL	25	Phase 2	Completed	61% ORR	The median PFS was 8.0 month; the median DOR was 20.3 months	NCT01998035
Romidepsin, Gemcitabine (158)	PTCL	20	Phase 2	Completed	30% ORR	Two-year OS rate was 50%, two-year PFS rate was 11.2%	NCT01822886
Romidepsin, Chidamide	AITL		Phase 2	Not yet recruiting			NCT04831710
Romidepsin, Azacitidine, Bendamustine, Gemcitabine	r/r AITL		Phase 3	Active, not recruiting			NCT03593018
Romidepsin	PTCL			Recruiting			NCT03742921
Romidepsin, Azacitidine, Belinostat, Pralatrexate, Gemcitabine	PTCL		Phase 2	Recruiting			NCT04747236
Romidepsin, Ixazomib	PTCL		Phase 1/2	Active, not recruiting			NCT03547700
Romidepsin, Pembrolizumab	r/r PTCL		Phase 1/2	Recruiting			NCT03278782
Romidepsin, Carfilzomib	r/r PTCL		Phase 1/2	Active, not recruiting			NCT03141203
Romidepsin, Lenalidomide	PTCL		Phase 2	Active, not recruiting			NCT02232516
Romidepsin, CHOEP	PTCL		Phase 1/2	Active, not recruiting			NCT02223208
Romidepsin, CHOP	PTCL		Phase 3	Active, not recruiting			NCT01796002
Romidepsin, Bortezomib, Duvelisib	r/r CTCL		Phase 1	Recruiting			NCT02783625
Romidepsin, Brentuximab vedotin	CTCL		Phase 1	Recruiting			NCT02616965
Romidepsin, Parsaclisib	r/r T-cell Lymphoma		Phase 1	Recruiting			NCT04774068
Romidepsin, Azacitidine, Duvelisib, Doxorubicin	T-cell Lymphoma		Phase 1	Not yet recruiting			NCT04639843
Romidepsin, Lenalidomide, Azacitidine, Dexamethasone	r/r T-cell Lymphoma		Phase 1	Recruiting			NCT04447027
Romidepsin, Azacitidine, Gemcitabine	T-cell Lymphoma		Phase 3	Active, not recruiting			NCT03703375
Romidepsin, Venetoclax	r/r T-cell Lymphoma		Phase 2	Recruiting			NCT03534180
Romidepsin, Carfilzomib, Lenalidomide	r/r T-cell Lymphoma		Phase 1/2	Active, not recruiting			NCT02341014
Romidepsin	T-cell NHL		Phase 2	Active, not recruiting			NCT01908777
Romidepsin, Lenalidomide	NHL		Phase 1/2	Active, not recruiting			NCT01755975
Panobinostat, Lenalidomide (159)	r/r HL	24	Phase 2	Completed	16.7% ORR	The median PFS was 3.8 months, the median OS was16.4 months	NCT01460940
Panobinostat, Ifosfamide, Carboplatin, Etoposide,	HL	40	Phase 1/2	Completed	85% ORR	65% Failure Free Survival	NCT01169636
Panobinostat, Everolimus	Lymphoma	61	Phase 1/2	Completed	33% ORR	20 mg panobinostat: the median PFS were 3.7 months; 30/40 mg panobinostat: the median PFS was 4.2 months	NCT00918333
Panobinostat, Rituximab	DLBCL	18	Phase 2	Terminated	11% ORR	The median PFS was 6 months	NCT01282476
Panobinostat, Rituximab (160)	DLBCL	40	Phase 2	Unknown status	28% ORR		NCT01238692
Panobinostat, Bortezomib (161)	PTCL	23	Phase 2	Completed	43% ORR	The median PFS was 2.59 months	NCT00901147
Panobinostat, Bexarotene (162)	CTCL	139	Phase 2	Completed	17.3% ORR	Bexarotene-exposed: the median PFS was 4.2 months; bexarotene-naïve: the median PFS was 3.7 months	NCT00425555
Panobinostat	r/r NHL	39	Phase 2	Active, not recruiting	21% ORR	The median PFS was 3.1 months, the median OS was 14.9 months	NCT01261247
Chidamide (163)	PTCL	79	Phase 2	completed	28% ORR	The median PFS was 2.1 months, the median OS was 21.4 months	
Chidamide, R-CHOP (164)	DLBCL	49	Phase 2	completed	94% ORR	Two-year PFS rate was 68%, two-year OS rate was 83%	NCT02753647
Chidamide (2021 ICML.Abstract No.209)	r/r PTCL	46	Phase 2	completed	46% ORR	The median PFS was 6 months, the medain OS was 23 months	
Chidamide, Cladribine, Gemcitabine, Busulfan (165)	r/r NHL	105	Phase 2	completed		Four-year PFS rate was 80.6%, four-year OS rate was 86.1%	NCT03151876
Chidamide, Sintilimab (2021 ASH Oral No.137)	ENKTL	30	Phase 2	completed	58% ORR, 47% CRR	The median PFS, OS, and DOR were 16.5, 28.5, and 20.6 months, respectively.	
Chidamide, Tislelizumab, Lenalidomid, Etoposide	r/r ENKTL	8	Phase 4	completed	87.5% ORR, 62.5% CRR		NCT04038411
Chidamide, Azacitidine	AITL		Phase 2	Not yet recruiting			NCT05179213
Chidamide	DLBCL		Phase 2	Recruiting			NCT04661943
Chidamide, Cyclophosphamide, Rituximab, Doxorubicin, Vincristine, Prednisone, Ibrutinib, Lenalidomide, Decitabine	DLBCL		Phase 2	Recruiting			NCT04025593
Chidamide, Rituximab, Gemcitabine,Oxaliplatin	r/r DLBCL		Phase 2	Recruiting			NCT04022005
Chidamide, Anti-PD-1 Antibody, Rituximab	r/r DLBCL		Phase 2	Not yet recruiting			NCT05115409
Chidamide	r/r B-cell NHL		Phase 2	Recruiting			NCT03245905
Chidamide, Decitabine, CD19/20 CAR-T cells	r/r B-cell NHL		Phase 1/2	Recruiting			NCT04553393
Chidamide, Cyclophosphamide, Doxorubicin, Vincristine, Prednisone	AITL		Phase 2	Recruiting			NCT03853044
Chidamide, Sintilimab	r/r AITL		Phase 2	Not yet recruiting			NCT04831710
Chidamide, Sintilimab, Azacitidine, L-DEP	ENKTL		Phase 2	Not yet recruiting			NCT05008666
Chidamide, Sintilimab	ENKTL		Phase 2	Not yet recruiting			NCT04994210
Chidamide	ENKTL-NT		Not Applicable	Recruiting			NCT04511351
Chidamide, Sintilimab	ENKTL		Phase 1/2	Recruiting			NCT03820596
Chidamide, Etoposide	NKTCL		Phase 4	Recruiting			NCT04490590
Chidamide, PD-1 Antibody, Lenalidomide, Etoposide	NKTCL		Phase 4	Recruiting			NCT04038411
Chidamide, PD-1 antibody, Peg-Asparaginase	NKTCL		Phase 2	Recruiting			NCT04414969
Chidamide, Sintilimab	r/r CTCL		Phase 2	Recruiting			NCT04296786
Chidamide, Cyclophosphamide, Doxorubicin, Vincristine, Etoposide, Prednisone	PTCL		Phase 2	Recruiting			NCT03617432
Chidamide, Azacitidine, CHOP	PTCL		Phase 3	Not yet recruiting			NCT05075460
Chidamide, Cyclophosphamide, Epirubicin, Vindesine, Etoposide, Prednisone	PTCL		Phase 1/2	Recruiting			NCT02987244
Chidamide, Azacitidine	PTCL		Phase 2	Recruiting			NCT04480125
Chidamide, PD-1 antibody	PTCL		Phase 2	Recruiting			NCT04512534
Chidamide, CHOP	PTCL		Phase 2	Recruiting			NCT04480099
Chidamide, Sintilimab, Azacitidine	r/r PTCL		Phase 2	Not yet recruiting			NCT04052659
Chidamide, Lenalidomide	r/r PTCL		Phase 2	Recruiting			NCT04329130
Chidamide, Parsaclisib	r/r PTCL		Phase 1/2	Not yet recruiting			NCT05083208
Chidamide, Mitoxantrone Hydrochloride Liposome Injection	r/r PTCL		Phase 3	Not yet recruiting			NCT04668690
Chidamide	Lymphoma		Phase 2	Active, not recruiting			NCT03629873
Chidamide, Camrelizumab, Decitabine	HL		Phase 2	Recruiting			NCT04233294
Chidamide, Decitabine, Camrelizumab, Decitabine, Camrelizumab	HL		Phase 2	Recruiting			NCT04514081
Chidamide, Decitabine, Camrelizumab	NHL		Phase 1/2	Recruiting			NCT04337606
Chidamide, Chiauranib	r/r NHL		Phase 1/2	Recruiting			NCT03974243
Chidamide, APG-1252	r/r NHL		Phase 1/2	Not yet recruiting			NCT05186012
Abexinostat	FL, MCL	30	Phase 1/2	Completed	FL: 56.3% (9/16) ORR; MCL: 21.4% (3/14) ORR		NCT00724984
Abexinostat	NHL		Phase 1/2	Recruiting			NCT04024696
Abexinostat, Ibrutinib	DLBCL, ML		Phase 1/2	Recruiting			NCT03939182
Abexinostat	r/r DLBCL		Phase 2	Recruiting			NCT03936153
Abexinostat	r/r FL		Phase 2	Recruiting			NCT03934567
Abexinostat	r/r FL		Phase 2	Active, not recruiting			NCT03600441
Entinostat (166)	r/r HL	49	Phase 2	Terminated	12% ORR, 24% DCR	The median PFS was 5.5 months, the medain OS was 25.1 months	NCT00866333
Entinostat, Pembrolizumab	r/r Lymphoma		Phase 2	Recruiting			NCT03179930
Entinostat, ZEN-3694	Lymphoma		Phase 1/2	Not yet recruiting			NCT05053971
Fimepinostat (167)	Lymphoma	33	Phase 1	Completed	24% ORR, 57% DCR		NCT01742988
Fimepinostat, Rituximab (168)	DLBCL	30	Phase 1	Completed	37% ORR	The medain DOR was 11.1 months, the median PFS was 2.9 months	NCT01742988
Fimepinostat (169)	r/r DLBCL and HGBL	66	Phase2	Completed	12% ORR, 30% DCR	The median PFS was 1.4 months	
Mocetinostat (170)	HL	51	Phase 2	Terminated	27% ORR		NCT00358982
Mocetinostat, Brentuximab Vedotin	r/r HL		Phase 1/2	Active, not recruiting			NCT02429375
Mocetinostat	r/r DLBCL, r/r FL		Phase 1/2	Active, not recruiting			NCT02282358
ITF2357, Mechlorethamine	HL	24	Phase 1/2	Completed			NCT00792467

##### 2.2.4.2 Belinostat

Belinostat is an isohydroxamic acid-derived pan-HDAC inhibitor that broadly inhibits all zinc-dependent HDAC enzymes (171). In a 2015 phase 2 study of relapsed/refractory PTCL (r/r PTCL), belinostat monotherapy demonstrated a completely durable response and manageable toxicity, showing an ORR in the 120 evaluable patients of 25.8% and a median PFS of 1.6 months (148). Based on this trial, belinostat monotherapy was approved by the FDA for the treatment of r/r PTCL patients in 2014. In a phase 2 trial for the treatment of r/r PTCL or relapsed/refractory CTCL (r/r CTCL), the ORR reached 25% in PTCL and 14% in CTCL (149). In a recent study, in patients with newly diagnosed PTCL, treatment with belinostat in combination with a standard cyclophosphamide, doxorubicin, vincristine, and prednisone (Bel-CHOP) regimen, resulted in an ORR of 86% (150). In addition, a randomized, phase 2B, multicentre, belinostat combination therapy trial for patients with r/r PTCL is recruiting (NCT04747236). The effect of belinostat in the treatment of B-cell lymphoma seems to be unsatisfactory. Among the 22 BL and DLBCL patients included, no patient achieved CR or PR (NCT00303953). The role of belinostat monotherapy or combination therapy in the treatment of B-cell lymphoma remains to be discussed. There is a phase 2 trial of belinostat as consolidation therapy with zidovudine for adult T-Cell leukemia-lymphoma (NCT02737046). Overall, the role of belinostat in the treatment of T-cell lymphoma is well established and its effectiveness in the treatment of B-cell lymphoma or other lymphoma needs to be further explored. Relevant clinical trials on belinostat are mentioned in **Table 3**.

##### 2.2.4.3 Romidepsin

Romidepsin is a potent and selective inhibitor of HDAC, arrests the cell cycle, induces apoptosis and inhibits angiogenesis by enhancing acetylation, both of histones and non-histones (172). In a phase 2 trial, 96 patients with CTCL were included who had received at least one or more systemic therapies. Of these 71% had an advanced disease (≥ 2B) (151). The primary endpoint ORR was 34%, including 6 patients with CR. 26 of 68 patients (38%) with advanced disease achieved remission, including 5 CR. The median response time was 2 months and the median DOR was 15 months. In addition, a clinically meaningful improvement in pruritus was observed in the trial with a median duration of pruritus reduction of 6 months. In a phase 2 study in patients suffering from CTCL, romidepsin treatment resulted in a clinically meaningful reduction in pruritus (CMRP). The clinical benefit was evaluated by using a patient-assessed visual analog scale. A total of 44 of 96 patients (46%) achieved a significant clinical benefit, including objective response and/or defined CMRP, and 43% of 73 patients with moderate-to-severe pruritus experienced CMRP. The median time to CMRP was 1.8 months and the median duration of CMRP was 5.6 months (173). Based on these two phase 2 trials, romidepsin was approved by the FDA in November 2009 for the treatment of r/r CTCL patients. Foss et al. studied the efficacy and safety of romidepsin in patients with r/r CTCL with tumor stage and folliculotropic mycosis fungoides, where patients received 14 mg/m^2^ of romidepsin on days 1, 8, and 15 of a 28-day cycle. The ORR to romidepsin treatment was found to be 45% (*n* = 20) in patients with skin tumors and 60% (*n* = 10) in patients with follicular disease involvement (149).

Two phase 2 studies examined the efficacy and safety of romidepsin in patients with PTCL. Of the 45 patients with PTCL included in the response analysis of the first study, 8 patients experienced CR and another 9 patients experienced PR with an ORR of 38% (152). The second phase 2 trial reported a 25% ORR, 15% CR/CR unconfirmed (CRu), a median of 1.8 months time to time to response (TTR), 17 months DOR (153). With a median PFS of 29 months, patients who achieved CR/CRu for ≥ 12 months had significantly longer survival versus those with CR/CRu for <12 months or <CR/CRu. For all patients, median PFS and OS were 4 months and 11.3 months, respectively (154). Based largely on these results, in 2011 the FDA accelerated approval of romidepsin for patients with ≥1 prior PTCL treatment. In a Japanese study of romidepsin in patients with r/r PTCL, the ORR was 43%, including 25% CR, with a median PFS of 5.6 months and a median DOR of 11.1 months (155). In a phase 3 trial comparing alisertib, gemcitabine, pralatrexate and romidepsin in patients with r/r PTCL, the ORRs were 33%, 35%, 43% and 43%, respectively, the median PFS were 115 days, 57 days, 101 days and 242 days, respectively (156). More studies are currently focusing on the combined use of romidepsin. Combined oral azacitidine and a Tfh phenotype showed a higher ORR (80%) and CRR (67%) (157). In a phase 2 study on the role of gemcitabine plus romidepsin (GEMRO regimen) in the treatment of r/r PTCL patients, the ORR was 30% with 15% CRR, the two-year OS rate was 50% and the two-year PFS rate was 11.2% (158). Another important finding is that a pretreatment regimen of romidepsin combined with busulfan and fludarabine reduces the risk of relapse after allo-SCT in patients with aggressive T-cell tumors(2021 ASH Oral No.553).

At present, most clinical trials are still concerned with the role of romidepsin in T-cell lymphoma. For AITL patients, there have been clinical trials with romidepsin in combination with chidamide (NCT03593018) and also with romidepsin in combination with azacitidine, bendamustine, gemcitabine (NCT03593018). More types of combination drug regimens are being explored in patients with PTCL, such as romidepsin in combination with azacitidine, belinostat, pralatrexate, and gemcitabine (NCT04747236), romidepsin in combination with ixazomib (NCT03547700), and romidepsin in combination with pembrolizumab (NCT03278782) etc. The treatment options being tried in CTCL patients are romidepsin united brentuximab vedotin (NCT02616965) and romidepsin united bortezomib and duvelisib (NCT02783625). The performance of romidepsin in B-cell lymphoma is still unknown and it is hoped that more clinical trials in this area will be seen in the future. Relevant clinical trials on romidepsin are mentioned in **Table 3**.

##### 2.2.4.4 Panobinostat

On February 23, 2015, the FDA approved panobinostat for the treatment of patients with multiple myeloma (MM). Panobinostat is a pan-HDACi with maximum potency against class I, II and IV histone deacetylases (174). The combination of panobinostat and the PI3K/mTOR inhibitor BEZ235 synergistically demonstrated effective inhibition of tumor growth and a prolonged survival in a mouse DLBCL xenograft model, demonstrating that PI3K inhibition enhances histone acetylation and enhances AKT dephosphorylation (175). In the trial of panobinostat and everolimus in the treatment of lymphoma, the combination treatment resulted in an ORR of 33% and the median PFS were 3.7 months and 4.2 months for patients treated with 20 mg and 30/40 mg panobinostat, respectively (NCT00918333). Panobinostat obtained a 21% ORR in NHL patients with a median PFS of 3.1 months (NCT01261247). In a phase 2 trial of panobinostat in combination with lenalidomide for the treatment of r/r HL, the ORR amounted up to 16.7%, which was lower than the ORR with either drug alone. The median PFS and OS were 3.8 and 16.4 months, respectively (159). In all 24 patients, grade 3 to 4 toxicities consisted of neutropenia (58%), throm-bocytopenia (42%), lymphopenia (25%), and febrile neutropenia (25%).These treatment results and adverse effects limited the further evaluation of this combination therapy. However, in a trial of panobinostat plus ifosfamide, carboplatin, and etoposide (ICE) against relapsed HL, the combination therapy demonstrated better results with an 85% ORR compared to ICE alone (75% ORR) (NCT01169636). Two clinical trials tested the efficacy of panobinostat in combination with rituximab in DLBCL. One of the trials was terminated due to slow accrual, and the results also showed that the combination therapy resulted in grade 3/4 thrombocytopenia in 44% of the patients (NCT01282476). The results of another trial were also unsatisfactory, with an overall remission rate of 29% for panobinostat and 26% for panobinostat plus rituximab. Moreover there appears to be a lacking benefit in adding rituximab to panobinostat (160).

In the treatment of T-cell lymphoma, panobinostat in combination with bortezomib performed well in the treatment of PTCL, reaching a 43% ORR (161). The greatest response was seen in patients with AITL, with 4 of 8 patients (50%) responding. Common treatment-related grade 3/4 AE continue to include thrombocytopenia (68%), and neutropenia (40%). In a CTCL setting, the benefit of a panobinostat with bexaroten combination therapy was greater than with panobinostat alone (20% ORR vs 15% ORR) (162). Clinical trials of panobinostat were stored in **Table 3**.

##### 2.2.4.5 Chidamide

Chidamide, an original anti-cancer drug with Chinese intellectual property rights, was approved for global marketing as a benzylamine histone deacetylase inhibitor, designed to block the catalytic pocket of class I HDACs and to inhibit the activity of HDAC1, 2, 3, and 10, which results in growth arrest and apoptosis. In a phase 2 study of r/r PTCL, 79 patients were treated with chidamide monotherapy (163). The results were significant, with an ORR of 28%, of these, 14% had CR/CRu. Patients with AITL tend to have a higher ORR (50%) and CR/CRu rates (40%), as well as longer lasting responses to chidamide therapy. The median PFS and OS were 2.1 and 21.4 months, respectively. The majority of AE were of grade 1/2 and those that occurred in ≥10% of the patients were of grade ≥3: thrombocytopenia (22%), leukopenia (13%), and neutropenia (11%). In 2017, Shi et al. published a paper describing chidamide in the treatment of r/r PTCL: a multicenter real-world study in China (176). For the 256 patients receiving chidamide monotherapy, the ORR and DCR were 39.06% and 64.45%, respectively, with a median PFS of 129 days. In 127 patients receiving chidamide in combination with chemotherapy, the ORR and DCR were 51.18% and 74.02%, respectively, with a median PFS of 152 days. In a phase 2 study of chidamide in combination with rituximab plus cyclophosphamide, doxorubicin, vincristine, and prednisone (R-CHOP) in 49 elderly patients with no reported grade 4 non-hematologic toxicity, suffering from newly diagnosed DLBCL, the CRR was 86%, ORR was 94%, and 2-year PFS and OS rates were 68% and 83%, respectively (164). These results suggest that chidamide in combination with R-CHOP is effective and safe in elderly patients with newly diagnosed DLBCL. A Japanese phase 2 clinical study demonstrates the effectiveness of chidamide monotherapy for r/r PTCL. The ORR was 46% in 46 evaluable patients and up to 88% in AITL patients. The median PFS and OS were 6 months and 23 months, respectively (2021 ICML.Abstract No.209). A new conditioning regimen with chidamide, cladribine, gemcitabine and busulfan (ChiCGB), significantly improved the outcome of high-risk or relapsed/refractory NHL (r/r NHL) (165). All 105 patients with high-risk, relapsed/refractory lymphoma who received ChiCGB as a conditioning therapy after transplantation of autologous peripheral stem cells, achieved complete hematopoietic recovery. At a median follow-up of 35.4 months, 80.6% of the patients were free of tumor progression with a high PFS rate and OS rate of 80.6% and 86.1%, respectively, with 94.5% of patients with B-cell NHL and 75.4% of patients with NKTCL surviving. Huang et al. initiated a clinical trial of sintilimab in combination with chidamide (SC) for the treatment of ENKTL. All patients first received 2-3 cycles of sintilimab (200 mg) in combination with chidamide (30 mg twice weekly). For early stage (stage I-II) patients, 2 cycles of SC combined with sequential 2-4 cycles of pegaspargase plus gemcitabine and oxaliplatin (P-Gemox) followed by involved field radiotherapy (IFRT) were given; for late stage (stage III-IV) patients, 3 cycles of SC followed by sequential 3-6 cycles of P-Gemox treatment were used. Of the 30 patients whose efficacy could be evaluated, the ORR reached 58%, with a CRR of 47%. The median PFS, OS and DOR were 16.5 months, 28.5 months, and 20.6 months, respectively. The incidence of AE was 56%, mainly from sintilimab or chidamide alone, and recovered by dose reduction or discontinuation, with the SC regimen demonstrating promising efficacy and high safety in patients with ENKTL (2021 ASH Oral No.137). Another regimen used to treat relapsed/refractory ENKTL (r/r ENKTL) is tislelizumab combined with chidamide, lenalidomid and etoposide. In 8 evaluable patients, 87.5% ORR and 62.5% CRR were achieved (NCT04038411).

A very large number of clinical trials have been conducted to explore the therapeutic effects of chidamide in different types of lymphoma. In patients with DLBCL, clinical trials are currently underway with chidamide monotherapy (NCT04661943), chidamide in combination with R-CHOP and placebo (NCT04231448), and chidamide in combination with rituximab, gemcitabine, and oxaliplatin (NCT04022005). In patients with AITL, the treatment options being tried are chidamide in combination with cyclophosphamide, doxorubicin, vincristine, and prednisone (NCT03853044), chidamide in combination with azacitidine (NCT05179213), and chidamide in combination with sintilimab (NCT04831710). Chidamide in combination with sintilimab has also been used in ENKTL patients (NCT05008666, NCT04994210). In contrast, in patients with NKTCL, more clinical trials have opted for a combination regimen with PD-1 antibodies (NCT04038411, NCT04414969). There are also many studies on how well chidamide works in patients with PTCL. These include not only combinations with traditional chemotherapy regimens such as CHOP (NCT05075460, NCT04480099), but also with other epigenetic drugs such as azacidine (NCT04480125), and with targeted drugs such as sintilimab (NCT04052659) and PD-1 antibodies (NCT04512534). In HL patients, the drugs of choice in most clinical trials are chidamide, decitabine and camrelizumab (NCT04233294, NCT04514081). Chidamide, an emerging HDAC inhibitor, has achieved good results in the treatment of certain types of lymphoma, and we need to further explore its efficacy for more types of lymphoma in the future. The trials mentioned above and other chidamide related trials are shown in **Table 3**.

##### 2.2.4.6 Abexinostat

Abexinostat (CRA-024781) is a broad-spectrum isohydroxamic acid-based HDAC inhibitor that demonstrated promising antitumor activity in a phase 1 clinical trial evaluating cancer (177). As shown in **Table 3**, the primary results reported in their phase 2 trial showed 56.3% ORR in FL and 21.4% ORR in MCL (NCT00724984). As there are fewer evaluations of the efficacy of abexinostat, more clinical trials are still focusing on the efficacy of abexinostat alone in the treatment of DLBCL (NCT03936153) and FL (NCT03934567, NCT03600441).

##### 2.2.4.7 Entinostat

Entinostat is a selective inhibitor of HDAC 1, 2, 3, and 11 (178). *In vitro* tests have shown that entinostat produces strong anti-proliferative and immunomodulatory signals through modulation of cytokine and chemokine levels, and displays synergistic effects when combined with immune checkpoint therapies (179, 180). In a phase 2 trial of entinostat against r/r HL, the ORR was 12% while the DCR was 24%, with a median PFS and OS of 5.5 and 25.1 months (166). Entinostat demonstrated good tolerability with significant clinical activity in a large number of pretreated HL patients. Based on these entinostat properties, combination applications with other drugs might be more promising in future trails. Clinical trials enrolling entinostat in combination with pembrolizumab (NCT03179930) or entinostat in combination with ZEN-3694 for lymphoma (NCT05053971). Relevant clinical trials mentioned are shown in **Table 3**.

##### 2.2.4.8 Fimepinostat

Recent evidence suggests that both the PI3K-Akt-mTOR signaling pathway and HDAC are effective targets in blood cancers. Dual targeting can overcome primary resistance and block secondary resistance due to compensatory/feedback mechanisms in cancer cells. Fimepinostat (CUDC-907) is also a dual inhibitor of HDAC (class I and II) and PI3K (class I α, β, and δ). In a phase 1 trial evaluating CUDC-907, single agent use reached 24% ORR. Of the 9 DLBCL patients, 2 patients achieved CR and 3 patients achieved PR, and SD was observed in 19 (57%) out of 37 patients evaluable for response (167). In its phase 1 expansion trial, 30 DLBCL patients were evaluated for CUDC-907 alone or in combination with rituximab (168). The results shown that the ORR of the evaluable patient cohort was 37%, with 9 of 19 (47%) reporting objective remission with monotherapy and 2 of 11 (18%) reporting objective response with combination therapy. The median PFS for all DLBCL patients in the study was 2.9 months, with a median PFS of 5.7 months in patients treated with monotherapy and 1.3 months in patients treated with combination therapy. In the phase 2 study that included 66 r/r DLBCL and high-grade B-cell lymphoma (HGBL) patients, CUDC-907 monotherapy amounted up to 12% ORR and the PFS was 1.4 months (169). However, monotherapy in patients with r/r DLBCL and HGBL in the presence of *MYC* alterations achieved an extended duration of reflection, and combination therapies or biomarker-based patient selection strategies may lead to higher response rates in future clinical trials. The tests mentioned above are listed in **Table 3**.

##### 2.2.4.9 Mocetinostat and Givinostat

Mocetinostat (MGCD0103) is an oral isotype-selective non-hydroxamic acid HDAC inhibitor targeting isotypes HDAC 1, 2, 3, and 11. It induces histone hyperacetylation, selectively induces apoptosis, and causes cell cycle arrest in a dose-dependent manner in various human cancer cell lines (181). In the phase 2 trial enrolling 51 patients with HL, the ORR for mocetinostat monotherapy was 27%, and 34 of 42 patients (81%) who completed at least 2 treatment cycles had decreases in tumor measurements (170). Another HDAC inhibitor against HL, givinostat (ITF2357), is also subject to clinical trials. These trails showed that ITF2357 in combination with mechlorethamine could achieve a 25% ORR and 28.66 months of PFS (NCT00792467). The above two drugs seem to have limited effect in the treatment of HL and hopefully in the future more epigenetic drugs will be available for the treatment of HL. There is now a clinical trial evaluating mocetinostat in combination with brentuximab vedotin in patients with HL (NCT02429375), and another study exploring mocetinostat alone in DLBCL and FL (NCT02282358). Relevant clinical trials mentioned are shown in **Table 3**.

#### 2.2.5 BET Inhibitors

As readers of histone acetylation, BET proteins can bind to acetylated lysine residues in the histone tail, thereby carrying the extended complex to the promoter region and activating transcription in that region. Histone acetylation is prevalent in super enhancers of oncogene expression, therefore inhibiting the binding of BET proteins to chromatin has a significant impact on transcription, which in turn resulted in the studies of many bromodomain inhibitors. The BET inhibitor RVX2135 has been shown in mouse models to inhibit lymphoma proliferation and to induce apoptosis. Moreover, it sensitizes *Myc* overexpressing lymphocytes by inducing HDAC silencing genes that synergize with HDAC inhibitors to kill lymphocytes (182). The small molecule inhibitor OTX015 (MK-8628) specifically binds to the bromodomain motif BRD2, BRD3, and BRD4 of BET proteins and keeps them bound to acetylated histones and this binding occurs preferentially in the oncogene super enhancer region. Based on a phase 1 trial evaluating the safety and pharmacokinetics of OTX015, the recommended once-daily dose of oral single-agent oral OTX015 in lymphoma patients was 80 mg, with an additional 9.1% ORR observed in 33 lymphoma patients (183).

### 2.3 Discussions

There is no doubt that the therapeutic effect of epigenetic drugs in lymphoma is remarkable. In the future, on the one hand we are interested in the appropriate dosing regimen in the treatment of lymphoma. Combining laboratory data with clinical experience provides the most beneficial recommendations for patients. Combining epigenetic therapies with other currently prevailing therapies, such as with immunotherapy, to combat refractory or relapsed lymphomas in a common face. On the other hand, the development of drugs for epigenetic interventions is undoubtedly promising and challenging, as systematic functional genomic and molecular mechanistic studies will provide new pathways and targets for “synthetic lethal strategies”; the development of computer-aided tools, animal models and other technologies will also create better conditions for lead compounds to enter clinical studies. Increased investment in research and development and larger screening scales will also accelerate the process of new drug development. We have every reason to believe that epigenetic therapies will fundamentally change the management of lymphoma patients and become an integral part of lymphoma treatment.

## Author Contributions

JXL, JNL, HW, and PL contributed to the conception and design of the study. JXL, JNL, and HW collected the data. JXL wrote the first draft of the manuscript. JNL and HW wrote parts of the manuscript. All authors contributed to the revision of the manuscript, read, and approved the submitted version.

## Funding

This work was supported by grants from the National Natural Science Foundation of China (Grant No. 81972595).

## Conflict of Interest

The authors declare that the research was conducted in the absence of any commercial or financial relationships that could be construed as a potential conflict of interest.

## Publisher’s Note

All claims expressed in this article are solely those of the authors and do not necessarily represent those of their affiliated organizations, or those of the publisher, the editors and the reviewers. Any product that may be evaluated in this article, or claim that may be made by its manufacturer, is not guaranteed or endorsed by the publisher.
